# Specimen preparation for X-ray micro-computed tomography of forest pests

**DOI:** 10.1186/s42649-025-00108-4

**Published:** 2025-04-02

**Authors:** Eun Jung Ahn, Ki Woo Kim

**Affiliations:** 1 Department of Ecology and Environmental System, Kyungpook National University, Sangju, 37224 Republic of Korea; 2 Animal & Plant Research Division, Nakdonggang National Institute of Biological Resources, Sangju, 37242 Republic of Korea; 3 Tree Diagnostic Center, Kyungpook National University, Sangju, 37224 Republic of Korea

**Keywords:** 3D imaging, Insect, Forest pest, Specimen preparation

## Abstract

X-ray micro-computed tomography (XCT) is an X-ray-based three-dimensional (3D) imaging technique that enables non-destructive imaging of both external and internal structures. It is widely used for studying biological specimens such as animals and plants. In this review, we discuss various specimen preparation methods for the technique, particularly focusing on forest pests, with six representative cases. Specimen preparation methods for forest pests can be broadly categorized into three groups based on mounting types: (i) simple mounting, (ii) liquid-cell mounting, and (iii) dry-cell mounting. The simple mounting method is particularly suitable for adult beetles due to their exoskeleton. The dehydration process minimizes specimen movement during scanning, ensuring better imaging quality. In the case of liquid-cell mounting, the specimen is immersed in a liquid medium for scanning, which effectively preserves the soft tissues of larvae and pupae. The dry-cell mounting does not involve fixation or dehydration and is particularly useful for analyzing immobilized specimens. To enhance the quality of 3D images, selecting an appropriate preparation method is essential. Since forest pests display varying sizes and types, the choice of preparation method should be based on the specific characteristics of the specimens of interest and research objectives. This review provides valuable insights for researchers and practitioners seeking to identify the most suitable and effective mounting method for XCT scanning of forest pests.

## Introduction

X-ray micro-computed tomography (XCT) is a powerful, non-destructive three-dimensional (3D) imaging technique that is similar to clinical computed tomography (CT) used in human medicine. While clinical CT resolutions are at the millimeter level, XCT can achieve micrometer-level resolutions (O’Sullivan et al. 2018), thereby delivering higher-resolution images (Du Plessis et al. 2017). Traditional sectioning is only able to provide 2D images, while virtual sectioning using XCT can generate various types of 3D images (Li et al. 2018). Due to these advantages, XCT is widely used for the histological analyses of animals and plants (Pajor et al. 2013; Pallua et al. 2015; Silvent et al. 2017).

The class Insecta is the taxonomic group with the greatest biological diversity (Sollai and Solari 2022). It is estimated that insects account for more than half of all animal species (Tihelka et al. 2021). Among the insect orders, Coleoptera, commonly known as beetles, represents approximately 40% of the identified insect species (Banerjee et al. 2014; Hunt et al. 2007). The number of Coleoptera species is estimated at approximately 400,000 (Hunt et al. 2007), with around 75% being phytophagous, feeding on plants and wood, which classifies them as pests (Gillott, 2005; Banerjee et al. 2014).

The application of XCT in insect research started in 2002. Compared XCT with traditional sectioning methods for studying the head of *Priacma serrata* (Hörnschemeyer et al. 2002). Their findings highlighted the convenience of XCT for analyzing the musculature of the head of *P. serrata*. In 2007, visualized the neural network of the brain of *Drosophila* by scanning larvae and producing comprehensive 3D images of their central nervous system (Mizutani et al. 2007). Following these advancements, laboratory XCT systems became more widely distributed, leading to an increase in morphological studies of insects. In 2017, first applied XCT to study forest pests, specifically Coleoptera, by examining the head of an adult poplar leaf beetle (*Chrysomela populi*), a species that feeds on various aspen trees (Ren et al. 2017). The scanned images provided detailed visualizations of their muscles and nervous systems.

Forest pests are one of the most significant threats to forest ecosystems worldwide, posing serious risks to biodiversity, timber production, and overall forest health (Guégan et al. 2023). Their impact has been exacerbated by climate change, which increased the frequency and severity of wood pest outbreaks by creating more favorable conditions for their proliferation and spread (Gely et al. 2020). Rising temperatures result in extended summers and warmer winters (Gely et al. 2020). During extended summer, elevated temperatures and humidity influence the fungal symbionts of insect vectors (Linnakoski and Forbes 2019). The aggressiveness of wood-boring insect pests is increasing, causing severe forest damage (Creyaufmüller et al. 2018). For scale insects, previous studies have shown that higher temperatures accelerate maturation and egg hatching (Frank 2021), which can increase tree mortality by shortening the generation times of forest insects (Ramsfield et al. 2016). Warmer winters enhance the overwintering survival of insect pests (Lawton et al. 2022), resulting in larger insect pest populations now inhabiting higher latitudes than in the past (Lawton et al. 2022; Simler-Williamson et al. 2019).

According to several studies, bark beetles, wood borers, and sap-sucking insects tend to thrive under dry conditions, taking advantage of the stress imposed on trees (Huberty and Denno 2004; Koricheva et al. 1998). During droughts, water stress leads to the accumulation of elevated nitrogen levels in tree organs, which can significantly alter their physiological state (Ramsfield et al. 2016). This increase in nitrogen levels enhances the nutritional quality of the trees for phytophagous insects, thereby facilitating their population growth and potentially triggering outbreaks of forest pests (Mattson and Haack 1987). Numerous studies have employed XCT to investigate ambrosia beetles, a type of wood-boring beetle (Li et al. 2018; Spahr et al. 2020), consistently demonstrating its high efficacy in analyzing their internal structures and organs (Li et al. 2018).

Appropriate specimen preparation is important for obtaining accurate 3D images. Biological specimens display varying sizes and shapes, thereby requiring different preparation methods. The five main steps in specimen preparation are as follows: (i) fixation, (ii) dehydration, (iii) staining, (iv) drying, and (v) mounting. Certain steps may be omitted depending on the sample type. This review discusses the characteristics of forest pest specimens and guides the preparation of various specimen types for XCT analysis based on six representative cases. This review aims to guide specimen mounting methods for XCT analysis of forest pests.

### General principles

**Fig. 1:**
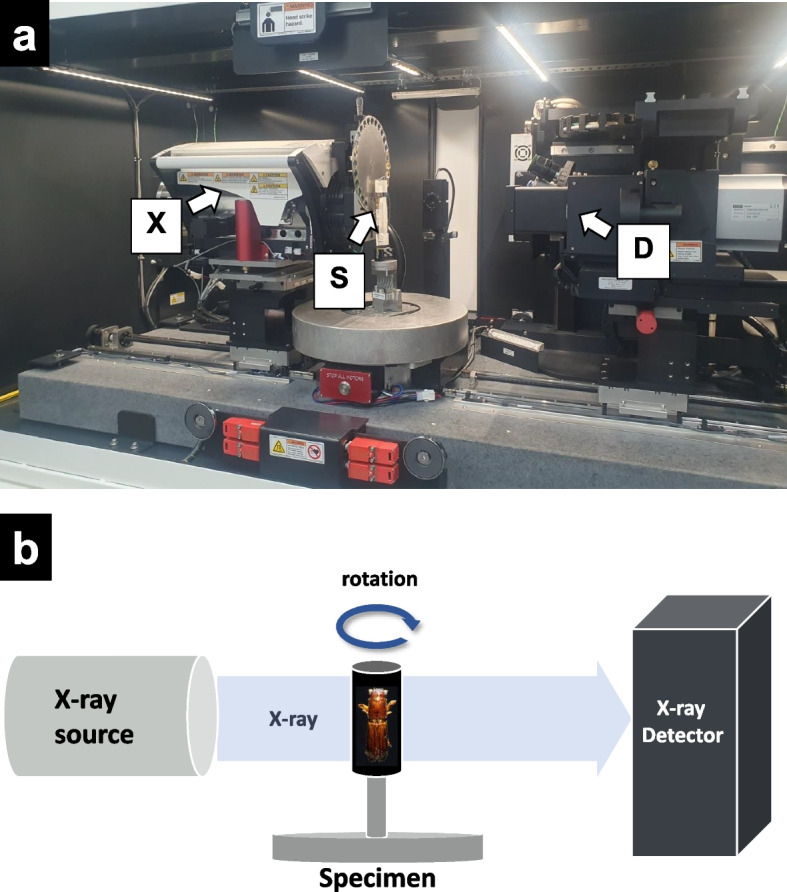
Representative images of X-ray micro-computed tomography (XCT). (a) The main components of XCT. X = X-ray source. S = specimen. D = X-ray detector. (b) A diagram illustrating the working principle of XCT

XCT is equipped with an X-ray source and a detector, with the specimen typically positioned between these two components (Fig. 1a). During the scanning process, the specimen is systematically rotated at a constant angle (Fig. 1b), enabling the capture of multiple projections for the reconstruction of detailed cross-sectional images. The purpose of specimen preparation for XCT is to (i) prevent structural changes, (ii) avoid movement during scanning, and (iii) enhance contrast. Each step of specimen preparation is intended to fulfill one or more of these objectives. Since insect specimens are often wet and fresh, fixatives like formalin and ethanol are necessary. Formalin induces the crosslinking of nucleic acids and proteins (Fox et al. 1985). Therefore, specimens preserved in formalin are not appropriate for DNA and protein assays (Chung et al. 2018). On the other hand, ethanol dehydrates proteins, causing shrinkage of specimens (Baker et al. 1958). It is critical to select an appropriate preservation method based on the specimen type.

As XCT is a non-destructive scanning method, it can be performed on live specimens. For in vivo XCT, the X-ray source and detector are rotated to adjust for possible specimen movements, while in vitro XCT only requires specimen rotation (Longo et al. 2017). Anesthesia is required to immobilize live samples for in vivo XCT, as XCT images are generated by stacking multiple 2D images. For the analysis of common laboratory animals such as mice, rats, and rabbits (Clark and Badea 2014), isoflurane is used for anesthesia (Wehrle et al. 2019). For insects, carbon dioxide (CO_2_) or sevoflurane is used (Poinapen et al. 2017; Lehmann et al. 2021).

Selecting the most appropriate mounting method is also important for specimen preparation where a non- or low-attenuating material is preferred for the sample holder (Vasconcelos et al. 2021).

Enhancing contrast is one of the main challenges in the XCT of biological specimens (Clark and Badea 2014). Soft tissues tend to yield low-contrast images (Clark and Badea 2014) and, therefore, require staining with a contrast agent to differentiate them from other organs. The most commonly used contrast agents are (i) osmium tetroxide, (ii) phosphotungstic acid (PTA), and (iii) iodine (Heimel et al. 2019). Osmium tetroxide can fix and stain unsaturated fatty acids (Belazi et al. 2009) and is commonly used for electron microscopy (EM) specimen preparation. PTA, and iodine-based contrast agents are used for their low toxicity (Swart et al. 2016).

**Fig. 2:**
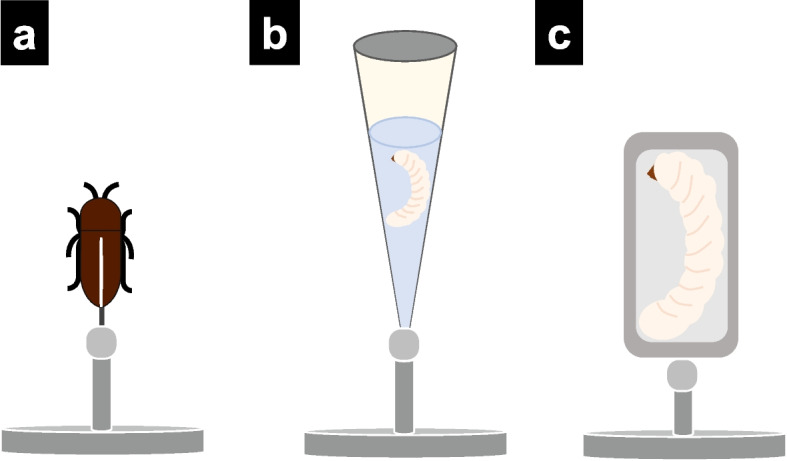
Diagrams of mounting types for XCT. **a** Simple mounting. **b** Liquid-cell mounting. **c** Dry-cell mounting

The drying step removes liquid from specimens (Runge et al. 2022) and improves image quality by enhancing the contrast of soft tissues. However, drying can cause structural damage to specimens (Sombke et al. 2015; Runge et al. 2022). During this step, a critical point drier (CPD) or hexamethyldisilazane (HMDS) is commonly used, similar to EM (Alba-Alejandre et al. 2019; Li et al. 2018). The specimens that are less susceptible to deformation, such as wood samples dried at room temperature, can be directly scanned (Park et al. 2020, 2022). In this review, we categorize specimen preparation for forest pests into three types of mounting: (i) simple mounting (Fig. 2a), (ii) liquid-cell mounting (Fig. 2b), and (iii) dry-cell method (Fig. 2c).

### Simple mounting

#### Case 1: Coffee borer beetle

The coffee borer beetle (*Hypothenemus hampei*) is the most significant pest of coffee trees (Jaramillo et al. 2011), with host specificity to coffee (Murphy et al. 1990). Economic losses caused by this pest result from primary damage to coffee seeds from boring and feeding (Damon., 2000). After an attack by adult female pests, the affected coffee seeds often suffer secondary infections caused by mycobiota (Carrión and Bonet 2004).

The size of the coffee borer beetle is around 1–2 mm in length, which makes it the smallest insect that can be analyzed by XCT (Alba-Tercedor et al. 2019). In several studies by Alba-Tercedor et al. specimens were prepared in different ways depending on the purpose of analysis (Alba-Alejandre et al. 2019; Alba-Tercedor et al. 2019).

**Fig. 3:**
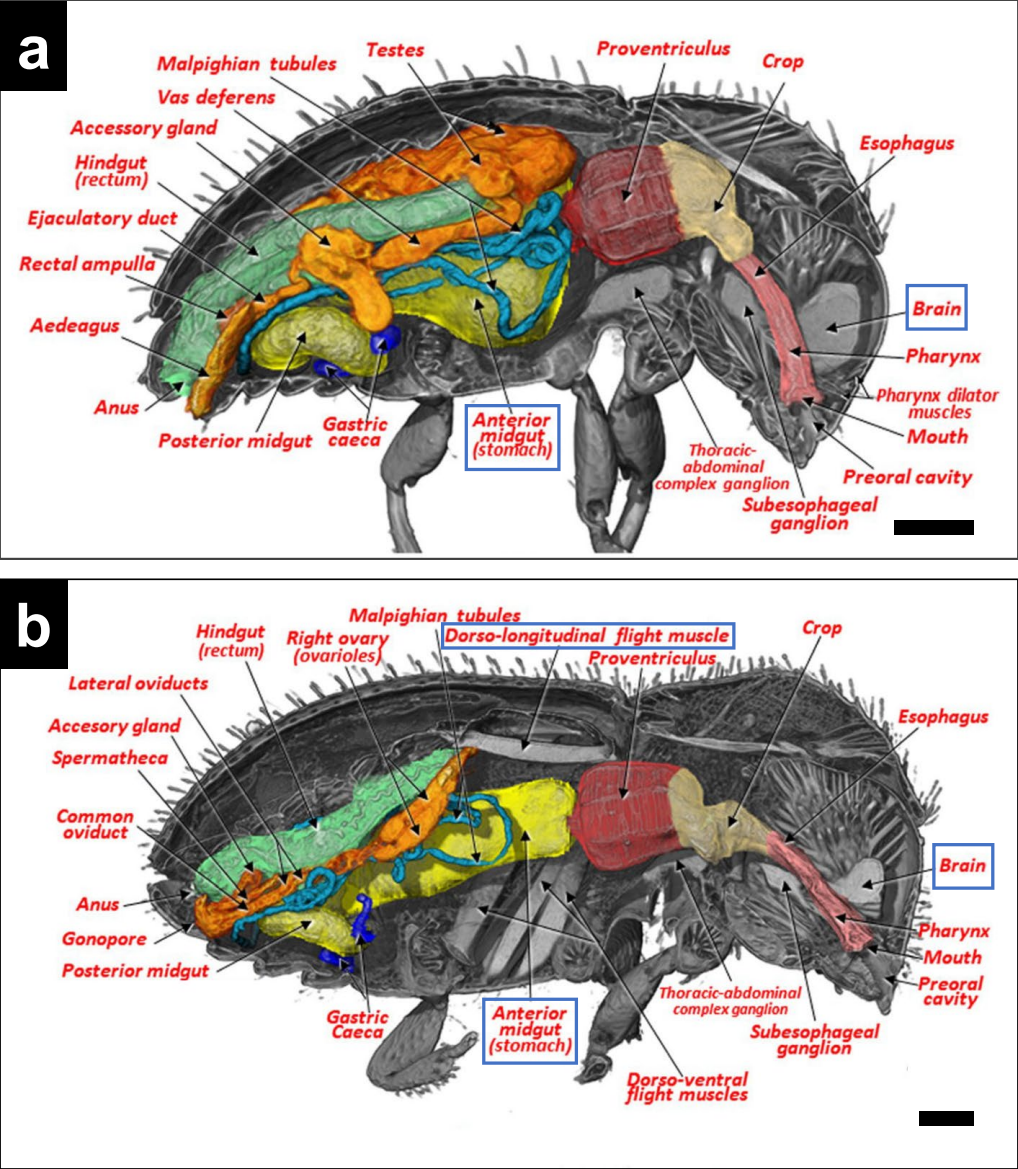
The internal anatomical analysis of the coffee borer beetle (*Hypothenemus hampei*) using XCT. **a** Lateral view of a male beetle. **b** Lateral view of a female beetle. Bars = 100 µm. Adapted from Alba-Alejandre et al. 2018, with permission from the publisher

For an anatomical study of sexual dimorphism of adult coffee borer beetles, male and female specimens were fixed in 30% ethanol for 25 min. They were dehydrated using a series of ethanol concentrations ranging from 50 to 90% (50, 70, 80, and 90%; 25 min each). The specimens were then stained with 1% iodine in anhydrous ethanol. The staining step was conducted for three days, after which the stained specimens were chemically dried in HMDS for four hours and then allowed to air-dry overnight. The specimens were then mounted on the tip of a fishing line. Detailed 3D images provided an anatomical view of the adult coffee borer beetle (Fig. 3a and b). The presence or absence of dorsal-longitudinal flight muscles is a key morphological characteristic that distinguishes their sexes. Only mature female adults can transfer from one seed to another for infection. Additionally, the size and shape of the brain and anterior midgut are also different between male and female coffee borer beetles. Their respiratory system was observed to have a diameter narrower than 1 μm. The obtained 3D images showed detailed structures of their tracheal systems.

**Fig. 4:**
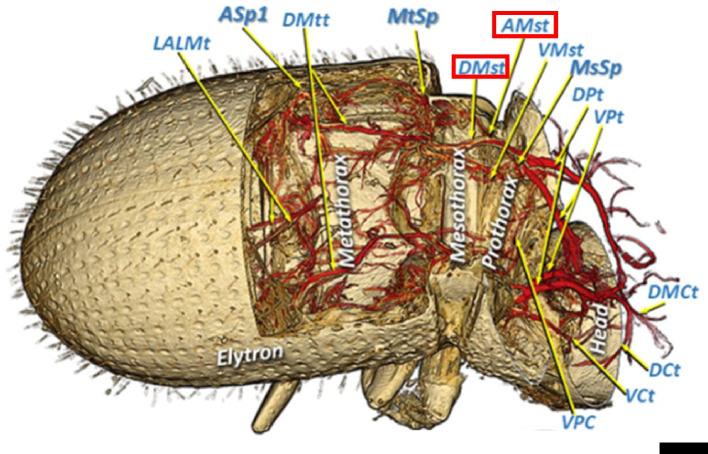
The sagittal section image of the tracheal tubes and spiracles of the coffee borer beetle (*Hypothenemus hampei*). AMst = arc-shaped mesothoracic trunk. DMCt = dorsal median cephalic trachea. Refer to the original article for more details. Adapted from Alba-Tercedor et al. 2019, with permission from the publisher

Additionally, adult female coffee borer beetles were exposed to ethyl acetate for 30 min. After death, the specimens were mounted on the tip of a fishing line. To compare their anatomical structures in fresh and dried conditions, the specimens were re-scanned after 14 months of air-drying. The obtained 3D images further revealed the tracheal systems of the coffee borer beetle (Fig. 4), where the tracheal tube in adult females was observed to be 70 times longer than their body length.

#### Case 2: Ambrosia beetles

Ambrosia beetles are wood-boring, fungus-farming beetles with approximately 3,500 species worldwide. They possess a unique organ called the mycangium for carrying symbiotic ambrosia fungi (Mayers et al. 2022). Ambrosia beetles do not consume wood but feed on fungi that infect the wood, thereby facilitating the transport of fungi (Huang et al. 2019).

The shape and location of mycangia vary depending on the species. To study them, Li et al. (2018) compared three visualization methods: (i) paraffin sectioning, (ii) laser ablation tomography (LATscan), and (iii) XCT. Five species of ambrosia beetles were scanned using micro-CT: *Ambrosiodmus minor, Euplatypus compositus*, *Premnobius cavipennis* *,* *Scolytoplatypus raja, Xylosandrus amputatus,* and *X. crassiusculus.*

**Fig. 5:**
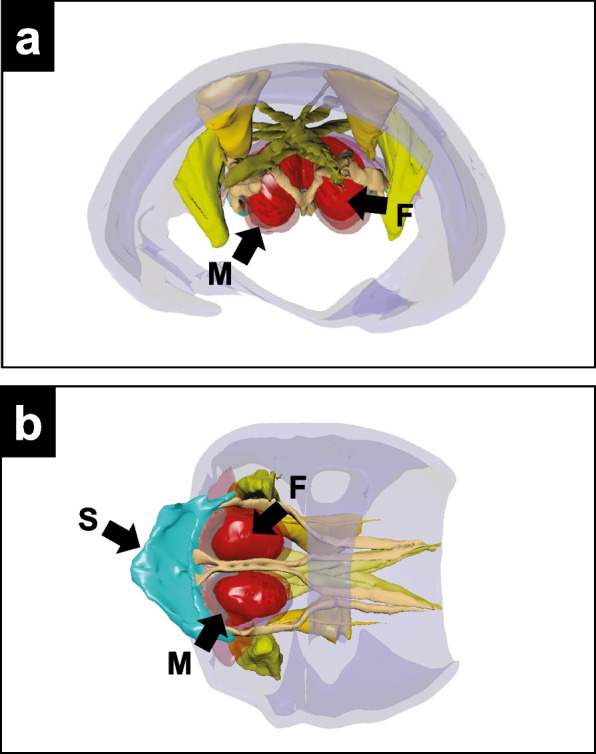
The 3D reconstruction images of mycangia and associated muscles of *Xylosandrus amputatus*. **a** Frontal view. M = mycangium. F = fungus. **b** Ventral view. S = scutellum. M = mycangium. F = fungus. Adapted from Li et al. 2018, with permission from the publisher

Here, the specimens were cryopreserved in ethanol and transferred to 95% ethanol. The specimens were dehydrated using a series of ethanol concentrations (95 to 100%). After dehydration, the specimens were dried using a CPD or at room temperature. This study highlighted XCT as the most efficient method for discovering new organs in ambrosia beetles. Virtual sectioning using XCT revealed different types of mycangia, yielding 3D reconstructed images of the mesonotal mycangium and associated muscles in *X. amputatus* (Fig. 5a and b)*.*

### Liquid-cell mounting

#### Case 1: *Euwallacea validus*

*Euwallacea validus* is a species native to East Asia, first reported in Japan and introduced to the United States in 1976 (Cognato et al. 2015). *E. validus* is a xyleborine ambrosia beetle that has a symbiotic relationship with fungi (Short et al. 2017). *E. validus* also has a pair of pre-oral mycangia inside its head (Berger 2017). *Fusarium oligoseptatum* *,* *Raffaelea subfusca,* and *Graphium* sp. are known symbionts of *E. validus* (Spahr, 2023). The host range of *E. validus* is broad, typically including declining or dead trees. However, an outbreak of *E. validus* could potentially lead to infections in healthy trees (Berger 2017).

**Fig. 6:**
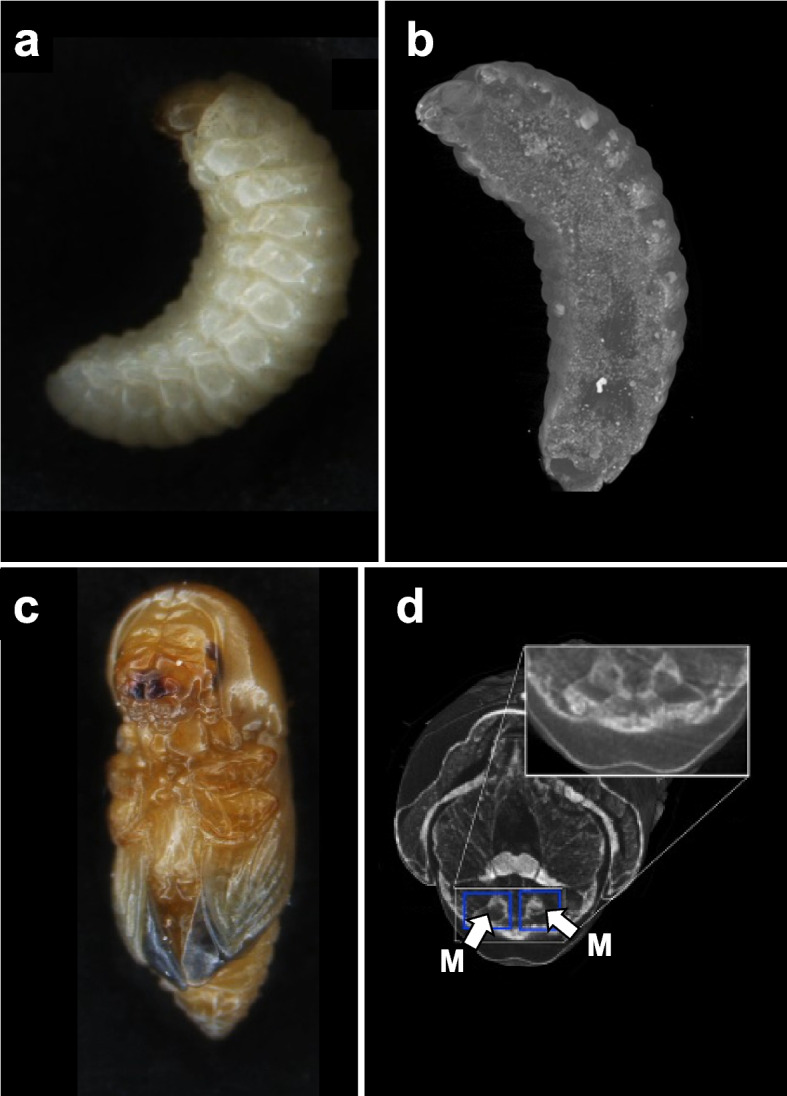
The external and internal features of *Euwallacea validus*. **a** Lateral view of the larva. **b** Virtual sagittal cross-section of the female larva. **c** Female pupa. **d** Virtual sagittal cross-sections of the female pupa. M = mycangium. Adapted from Spahr et al. 2020, with permission from the publisher

In a study by Spahr et al. (2020), the development of *E. validus* mycangia were examined in male and female larvae, pupae, and adults (Fig. 6a-d). The mycangia of *E. validus* develop in late-stage pupae in females, whereas male pupae do not exhibit a mycangial boundary at the same stage. Additionally, Spahr et al. (2023) examined the functions of specific genes related to the development of *E. validus* mycangia using gene knockdown of *breathless* (*btl*), *trachealess* (*trh*), and *doublesex*. XCT visualization revealed that the development of major mycangia is impaired in insects with *btl* and *trh* gene knockdowns.

Here, all specimens were fixed in 70% ethanol for more than one night. The specimens were then stained overnight with an iodine-based chemical (5% Lugol’s solution). Using this method, the specimens could be mounted without drying. The specimens were then immersed in ethanol and placed in 200-μL pipette tips, which served as sample holders.

#### Case 2: Harlequin ladybird

The harlequin ladybird (*Harmonia axyridis*), also known as the Asian ladybird, is native to Korea, Japan, China, Mongolia, Vietnam, and Siberia (Brown et al. 2008; Roy et al. 2016). It is widely recognized as a natural predator of aphids and has been intentionally released in North America and Europe as a biological control agent (Koch and Galvan 2008). Unexpectedly, such introduction of the harlequin ladybirds to the respective regions has led to significant adverse outcomes, as they feed not only on target aphids but also on non-target insects and plants, particularly grapevines (Koch and Galvan 2008; van Lenteren et al. 2008). The harlequin ladybirds have been observed to consume undamaged grape berries (Koch et al. 2004). With its broad habitat range, they are now one of the most widely distributed ladybird species globally. Although they are still considered a regional pest, they have the potential to become a global invasive species (Roy et al. 2016).

**Fig. 7:**
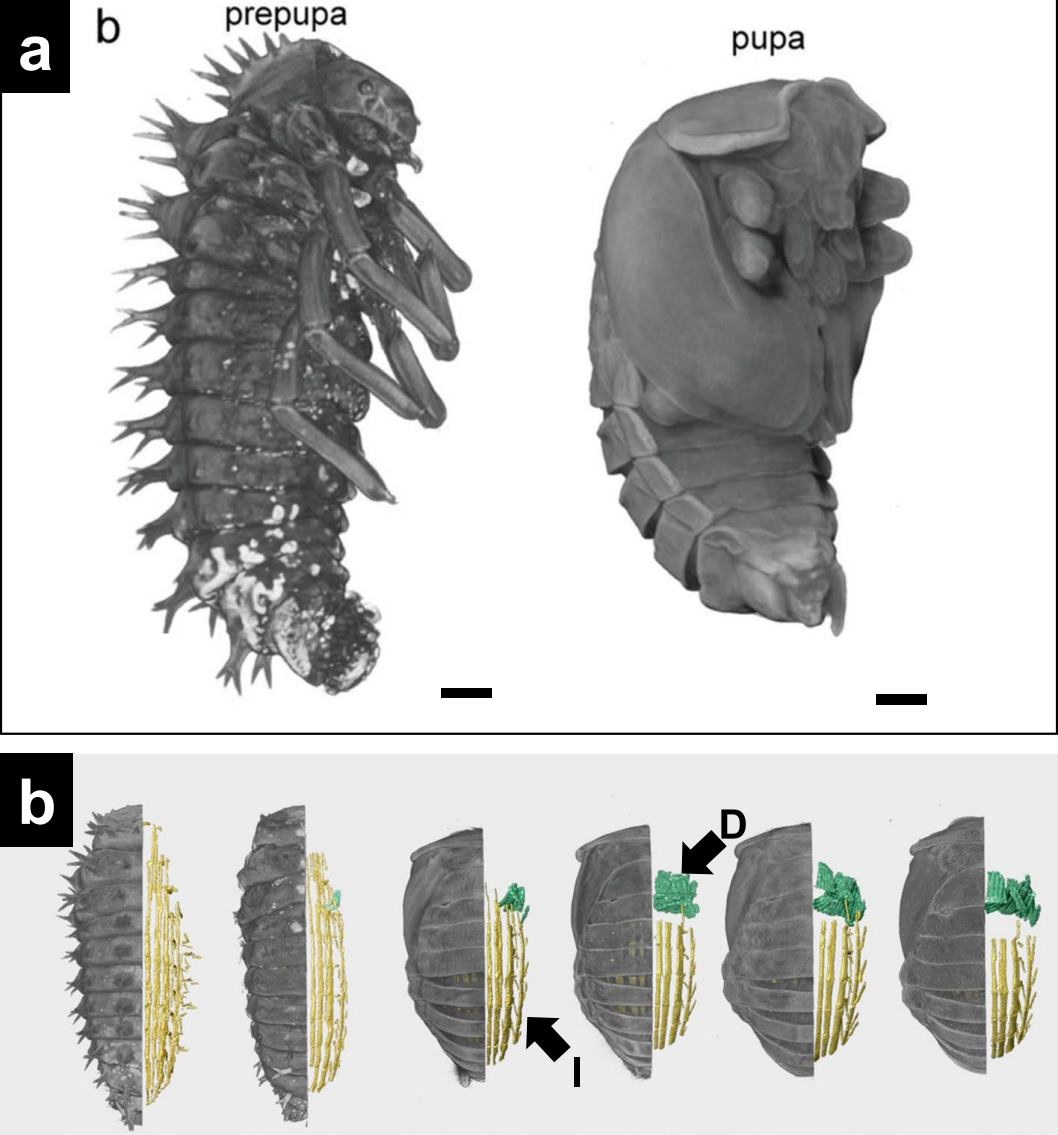
The 3D virtual analysis of pre-pupa and pupa of harlequin ladybird (*Harmonia axyridis*). **a** 3D models of the pre-pupa and pupa. Bar = 100 µm. **b** Lateral view showing muscle changes during metamorphosis. I = indirect flight muscle. D = direct flight muscle. Adapted from Shu et al. 2024, with permission from the publisher

In a study by Shu et al. (2024), 3D models of the prepupa and pupa stages of the harlequin ladybirds were generated to investigate the changes in internal organs during metamorphosis. The insects were incubated under laboratory conditions at 25 ± 1 °C and 75% relative humidity. During the fixation step, the specimens were anesthetized with CO_2_ and subsequently preserved in 75% ethanol. After 24 h, all specimens were washed three times with phosphate-buffered saline (PBS). The staining process lasted one week, during which 1 mL of standard Lugol’s solution was applied. After staining, the specimens were washed three times with PBS and stored at 25 °C. For scanning, 200-μL pipette tips were used as sample holders. The tops of the tips were trimmed and sealed at high temperatures. Subsequently, the tips were filled with PBS and sealed with parafilm to prevent drying. The obtained 3D reconstruction images showed major internal changes in *H. axyridis* from pre-pupa to pupa. For example, the indirect flight muscles were observed in the dorsal plate during the late prepupal stage (Fig. 7a and b).

### Dry-cell mounting

#### Case 1: Coffee borer beetle

Coffee borer beetles cause significant damage as adult females bore into immature and mature coffee seeds to oviposit (Damon, 2000). Almost every life cycle of these beetles occurs within coffee seeds (Johnson et al. 2020). Adult females can colonize new seeds once they are mature, whereas males remain in the initially infected seeds for their whole life cycle (Alba-Alejandre et al. 2018).

**Fig. 8:**
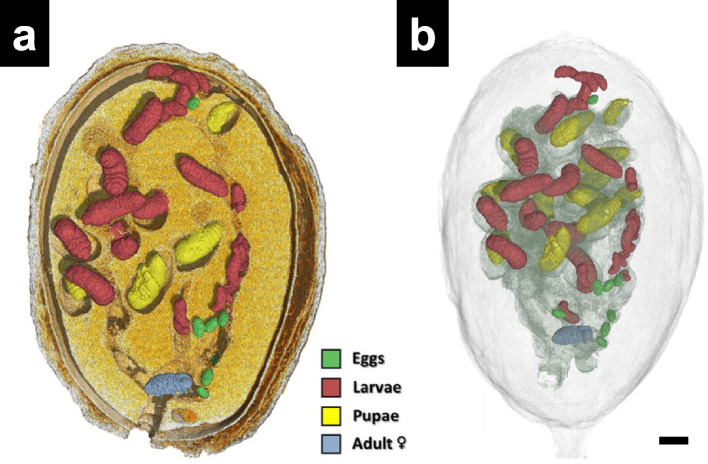
The internal features of an infested coffee seed. **a** The meso-sagittal section of coffee seeds infested by the coffee borer beetles (*Hypothenemus hampei*). **b** Digitally transparent images with the coffee seeds removed. Bar = 1 mm. Adapted from Alba-Alejandre et al. 2018, with permission from the publisher

XCT has been used to observe these beetles within the seeds of *Coffea canephora* collected in Vietnam in 2017. The invaded coffee seeds were stored at room temperature, and the specimens were mounted on melamine foam in a plastic container. Ethyl acetate was dropped onto the melamine foam for sample immobilization. The volume-rendered images showed different developmental stages of the beetles (eggs, larvae, pupae, and female adult) along with galleries inside the invaded coffee seeds in intact forms (Fig. 8a and b). The insets were segmented by developmental stages within the infected seeds.

#### Case 2: *Cacosceles newmannii*

*C. newmannii* is a large wood-boring beetle native to Mozambique, Eswatini, and South Africa (Ferreira 1980). The family Cerambycidae are forest insects that decompose wood, with certain species posing significant threats to economically important forests (Haack et al. 2010; Javal et al. 2019). The host plants of *C. newmannii* include woody plants, particularly those in the Myrtaceae family (Ferreira 1980). The host range of *C. newmannii* was poorly studied until 2015 (Smit et al. 2021; Way et al. 2017), when it was identified as a new pest of sugarcane in South Africa, posing a significant threat to the sugarcane industry. Studies on its outbreaks and associated impacts are urgently required (Way et al. 2017).

**Fig. 9:**
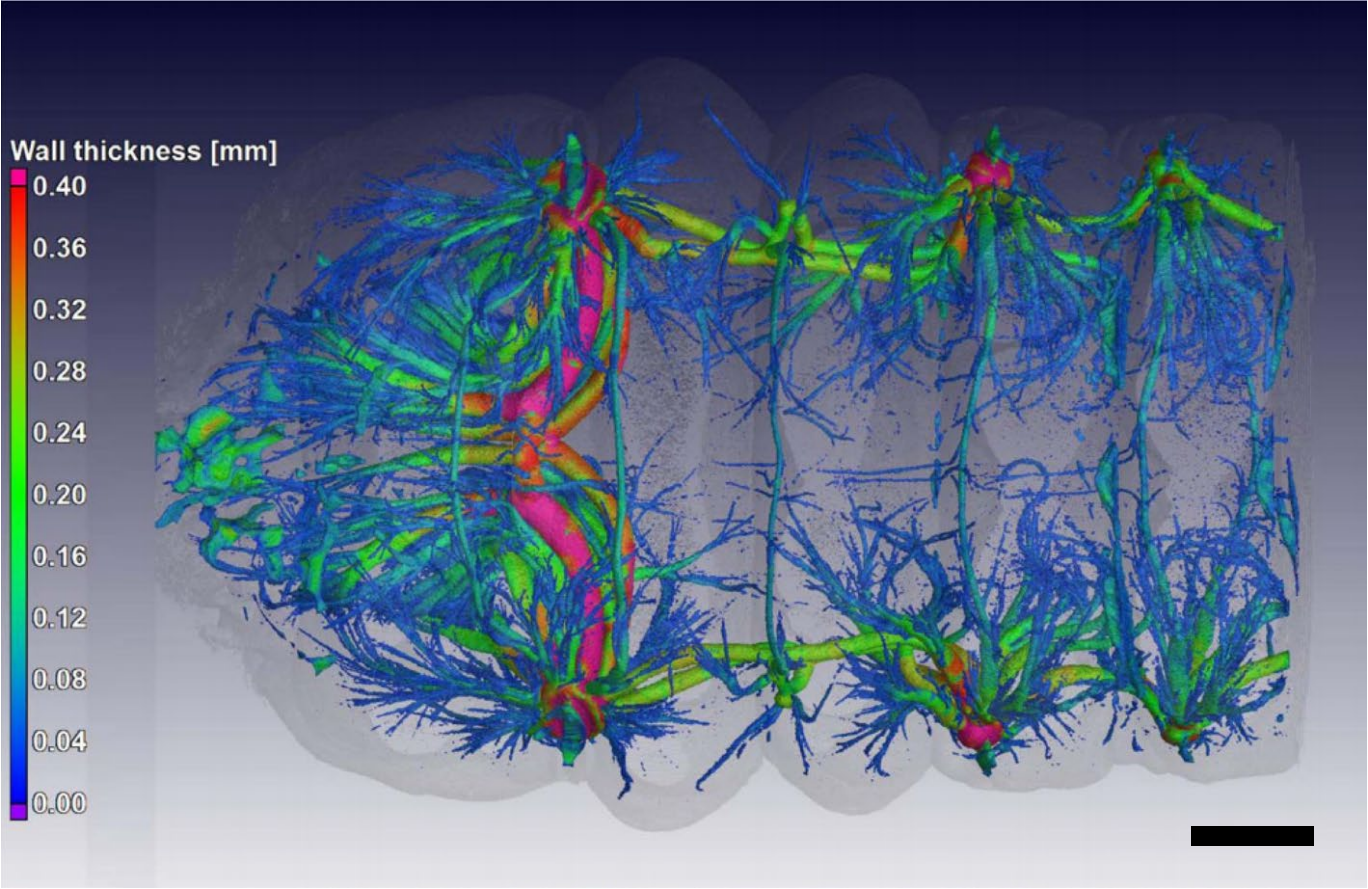
The 3D model of the tracheal system of live *Cacosceles newmannii* larvae. Differences in tracheal channel thickness are represented through color coding. Bar = 2 mm Adapted from Lehmann et al. 2021, with permission from the publisher.

The 3D visualization and quantification of the tracheae of *C. newmannii* have been conducted by Lehmann et al. (2021) through repeated scans of live larvae. Here, the larvae were put into 15-mL Falcon tubes packed with cotton, to which 100–200 μL of sevoflurane was added for anesthesia. A closed-cell foam was used for mounting the larvae. After scanning, the larvae were removed from the tubes and allowed to recover, regaining consciousness within 60 s. Using 3D reconstruction images of these live specimens, the tracheal structure of *C. newmannii* could be segmented (Fig. 9).

## Conclusions and outlook

In this review, methods for preparing forest pest specimens were classified into three types. Simple mounting is commonly performed on specimens after drying and is applied to adult beetles due to their hard exoskeleton. In the case of liquid-cell mounting, specimens are fixed in preservation liquid and scanned while still submerged. This method is suitable for pupae, larvae, and adult beetles, as it facilitates the analysis of soft tissues. The dry-cell method is used for immobilized live or freshly collected specimens that require anesthesia or euthanasia. When specimens become damaged or dehydrated during scanning, scan times must be shortened.

For analyzing forest pests, embedding methods may be beneficial, particularly for those with significant soft tissues, such as larvae or pupae. These methods are often utilized for studying soft tissues in vertebrates, with acrylic resin embedding specifically applied in XCT studies of zebrafish (Katz et al. 2021). Additionally, XCT has been integrated with transmission electron microscopy for analyzing human lung tissues for its non-destructive capabilities (Morales et al. 2016). Recently, next-generation ultramicrotome systems have been developed to enable trimming in conjunction with XCT data (Meechan et al. 2022; Micheva et al. 2024). The combined use of XCT with EM at the specimen preparation stage may be highly beneficial. Additionally, XCT has the potential to be utilized for studying parasites associated with forest pests. Van De Kamp et al. (2018) employed synchrotron X-ray tomography to investigate parasitoid wasps within fly pupae preserved in mineralized fossils. Since parasitoid wasps are often used for the biological control of forest pests, their relationship may be effectively studied using XCT (Wang et al. 2019).

Moreover, one of the most significant advantages of XCT is its ability to image live samples. While safety tests on live laboratory animals have been conducted, research on invertebrates remains limited. In the future, methods for preparing live specimens for XCT analysis must be further explored, particularly for studies involving forest pests.

## Data Availability

Data and materials available on request.

## Abbreviations

XCT: X-ray micro-computed tomography
3D: Three-dimensional
2D: Two-dimensional
DNA: Deoxyribo nucleic acid
PTA: Phosphotungstic acid
EM: Electron microscopy
CPD: Critical point drier
HMDS: Hexamethyldisilazane
LATscan: Laser ablation tomography
